# Serum vitamin D receptor and High Mobility Group Box-1 (HMGB1) levels in HIV-infected patients with different immunodeficiency status: A cross-sectional study

**DOI:** 10.1016/j.amsu.2021.02.020

**Published:** 2021-02-12

**Authors:** Indah Sapta Wardani, Mochammad Hatta, Risna Halim Mubin, Agussalim Bukhari, Mulyanto, Muhammad Nasrum Massi, Irawaty Djaharuddin, Burhanuddin Bahar, Aminuddin, Siti Wahyuni

**Affiliations:** a Post Graduate School, Faculty of Medicine, Hasanuddin University, Makassar, Indonesia; b Department of Internal Medicine, Faculty of Medicine, Mataram University, Mataram, Indonesia; c Department of Microbiology, Faculty of Medicine, Hasanuddin University, Makassar, Indonesia; d Department of Internal Medicine, Faculty of Medicine, Hasanuddin University, Makassar, Indonesia; e Department of Nutritional Sciences, Faculty of Medicine, Hasanuddin University, Makassar, Indonesia; f Department of Imunobiology and Laboratory, Faculty of Medicine, Mataram University, Mataram, Indonesia; g Department of Pulmonology, Faculty of Medicine, Hasanuddin University, Makassar, Indonesia; h Department of Nutrition, Faculty of Public Health, Hasanuddin University, Makassar, Indonesia; i Department of Parasitology, Faculty of Medicine, Hasanuddin University, Makassar, Indonesia

**Keywords:** Vitamin D receptor, HMGB1 protein, HIV infection, Immunodeficiency

## Abstract

**Background:**

HIV-AIDS patients typically have hypovitaminosis D. Vitamin D is a key mediator in inflammatory and infectious diseases, which VDR mediates its biological effect. High-mobility group box 1 protein (HMGB1) modulates HIV-1 replication in vitro. Vitamin D played a role in inhibiting HMGB1 secretion in the animal study.

**Objectives:**

This study aimed to examine differences and correlation of vitamin D receptor and HMGB1 protein levels in HIV patients with mild and severe immunodeficiency and healthy control participants.

**Methods:**

This study using a cross-sectional design conducted at Volunteer Counseling and Testing (VCT) Clinic in Mataram, West Nusa Tenggara, Indonesia, from January to June 2020. Three groups of study participants were classified as HIV patients with severe immune deficiency (SID), HIV patients with mild immune deficiency (MID), and healthy controls (HC).

**Results:**

Mean level of vitamin D receptor in SID HIV group was 25.89 ± 3.95 ng/ml, lower than those in MID-HIV group; 33.72 ± 1.69 ng/ml and in HC group; 50.65 ± 3.64 ng/ml. Mean levels of HMGB1 protein in the SID HIV group were 3119.81 ± 292.38 pg/ml higher than those in the MID HIV group 1553.55 ± 231.08 pg/ml and HC 680.82 ± 365.51 pg/ml. There was a significant and strong negative correlation (r = −0.932) between vitamin D receptor and HMGB1 levels (*p* < 0.01).

**Conclusions:**

Strong negative correlation between VDR and HMGB1 in different immunodeficiency statuses suggesting an important role of vitamin D in inflammation control in HIV infection. However, it needs to be confirmed in a further prospective study.

## Highlights

• Study was conducted in developing area in West Nusa Tenggara, Indonesia.
• This study consists of 3 groups with different immune status.
• This was the first study that correlates VDR and HMGB1 in HIV-infected patients.
• Strong correlation suggests association of VDR and HMGB1with HIV infection.

## 1 Introduction

HIV remains a global health problem with increasing incidence worldwide. At the end of 2019, an estimated 38 million people lived with HIV, 1,7 million new infections, and caused 690,000 AIDS-related deaths globally [1]. In Indonesia, the cumulative number of people with HIV in 2017 reached 280,623 people, with those diagnosed with AIDS reaching 102,667 people [2]. In West Nusa Tenggara, until December 2018, the cumulative number of cases was reported 812 HIV cases and 937 AIDS cases [3].

Chronic inflammation and immune activation are still the causes of morbidity and mortality in people with HIV, even though they have received ARV therapy [4]. People living with human immunodeficiency virus (HIV) infection typically have hypovitaminosis D, which is linked to many pathologies, including immune disorders and infectious diseases [5]. Chronic inflammation decreases vitamin D production, which is a key mediator in inflammatory and infectious diseases [6]. The biological effect of vitamin D is mediated by VDR and related to VDR protein expression level. Vitamin D and VDR axis have a strong immunomodulatory effect and antimicrobial effect in HIV infection [7].

High mobility group box 1 (HMGB1) is a protein that has been associated with inflammatory processes. HMGB1 contributes to immune activation and HIV-related progressivity [8]. HMGB1 induces HIV replication in vitro [9]. The passive release of HMGB1 causes excessive activation of the immune system in HIV patients [10]. Previous research in the animal study showed that vitamin D played a role in inhibiting HMGB1 secretion [11].

Several studies have reported the incidence of vitamin D insufficiency in people with HIV, association with inflammation, and their importance during HIV infection [7]. However, no studies have been conducted on vitamin D receptor and HMGB1 levels and their association with the grading of immune status in HIV patients. This reason leads the current study to examine the correlation and differences in vitamin D receptor and HMGB1 protein levels with the immune status of HIV patients.

## 2 Methods

This study used a cross-sectional design conducted at the VCT clinic in Mataram (West Nusa Tenggara) Indonesia from January to June 2020. Three groups of 39 study subjects consisting of 13 participants of HIV patients with severe immune deficiency (SID), 13 participants of HIV patients with mild immune deficiency (MID), and 13 healthy controls (HC). Study participants were selected with purposive sampling and diagnosed with HIV positive serology, treated and recorded during the January–December 2019 period, which met inclusion and exclusion criteria. Inclusion criteria were; 1. Patients with HIV who were diagnosed serologically as HIV positive; 2. Over 18 years of age; 3. Completed medical record data; 4. Routine control at the VCT clinic in Mataram; 5. HIV patients who received treatment of one of ARV combinations according to the Ministry of Health guidelines and signed consent letter. The exclusion criteria were as follows: 1. Pregnant, severe kidney disease, severe liver problems; 2. HIV patients who drop out of medication; 2. Patients on therapy or using vitamin D supplementation; 4. Patients with BMI <16 kg/m^2^. BMI was determined according to Asian standard i.e., Low BMI= <18.5; Normal BMI = 18.5–22.9; High BMI = ≥23.

Data were collected from direct blood sampling and data collection from medical records. The medical record included patient profile (name, medical record number, age, weight, date of birth, gender, address, education, occupation, height, and weight), basic hematology laboratory results, kidney function, liver function, CD4 count data, ARV data used, and data from questionnaires on sun exposure, lifestyle, and diet. Examination of vitamin D receptor and HMGB1 protein levels were carried out at the Laboratory of Molecular Biology and Immunology, Medical Faculty of Hasanuddin University Makassar. The research has been registered at www.researchregistry.com with research registry UIN:researchregistry6352. The research has also been approved by the Research Ethics Committee of Faculty of Medicine, Hasanuddin University with Reference No. 1209/UN4.6.4.5.31/PP36/2019.

Serum vitamin D receptor and HMGB1 protein levels were examined in duplicate using ELISA method (Vitamin D receptor: LSBio LS-F27316 and HMGB1: LSBio LS-F4038). Reading was performed at a wavelength of 450 nm.

Obtained data were processed using SPSS version 23.0. The parameters and characteristics of the samples were described in three groups. Data were expressed as mean and standard deviation for vitamin D receptor and HMGB1 protein serum levels. The normality test was carried out using the Shapiro Wilk, followed by a non-parametric test with Kruskal Wallis with a level of significance of *p* < 0,05. Spearman's correlation was used to determine the correlation between vitamin D receptor and HMGB1 protein levels with immune status. This study was conducted following the STROCSS criteria [12].

## 3 Result

Thirty-nine study's participants were divided into three groups including 13 HIV patients with severe immune deficiency (SID), 13 HIV patients with mild immune deficiency (MID), and 13 healthy controls (HC). The baseline characteristic of the study participants is shown in Table 1.

**Table 1:** Baseline characteristics of the study groups.

Baseline characteristic	HC n = 13	MID-HIV n = 13	SID-HIV n = 13	*p-*value
**Age**	33,85 ± 7,046	35,69 ± 9,25	35,08 ± 11,18	0.922
**Sex**				
1. Male	4 (10,2%)	8 (20,5%)	9 (23,07%)	0.121
2. Female	9 (23,07%)	5 (12,8%)	4 (10,2%)	
**Education**				
1. Uneducated/Primary School	–	1 (2,5%)	1 (2,5%)	0.024
2. Secondary school	–	5 (12,8%)	–	
3. High school	5 (12,8%)	4 (10,2%)	7 (17,9%)	
4. Diploma/Bachelor	8 (20,5%)	3 (7,6%)	5 (12,8%)	
**Employment**				
1. Unemployed/Housewives/College student/Retired	4 (10,2%)	6 (15,3%)	5 (12,8%)	0.289
2. Private employee	4 (10,2%)	6 (15,3%)	7 (17,9%)	
3. Civil servant/Police/Teacher/Lecturer	5 (12,8%)	1 (2,5%)	1 (2,5%)	
**BMI**				
1. Low BMI	–	–	6 (15,3%)	0.006
2. Normal BMI	13 (33,3%)	13 (33,3%)	7 (17,9%)	
3. High BMI	–	–	–	
**Comorbidities**				
1. Hypertension		–	2 (5%)	0.128
1. Diabetes Mellitus	–	1 (2,5%)	–	0.368
2. Hepatitis B	–	–	1 (2,5%)	0.368
3. Renal Insuficiency	–	–	1 (2,5%)	0.368
4. Cardiovascular Disease	–	–	2 (5%)	0.128
5. Anemia	–	–	6 (15.3%)	0.001

HC: Healthy Control, MID-HIV: Mild Immunodeficiency-HIV patients, SID-HIV: Severe Immunodeficiency-HIV patients, HIV: Human Immunodeficiency Virus, BMI: Body Mass Index.

In this study, all patients (100%) were in productive age, with a minimum age of 18 years and maximum age of 54 years. The mean age of the study subjects in the HIV group was 35.4 years. Based on gender, the subjects were 21 men (53.8%) and 18 women (46.1%). The highest education levels were high school and diploma, as many as 16 people (41.02%). Most participants in each group were employees (61.4%), and the rest were unemployed (38.4%). Nutritional status based on BMI was 84.6% in a normal range of the total study participants, especially in the HIV group, there was 77% with normal BMI and 23% with low BMI. The comorbid conditions including anemia 10%, cardiovascular disease 5%, hypertension 5%, DM 2.5%, hepatitis B 2.5% and renal insufficiency 2.5%.

The profile of serum vitamin D receptor and HMGB1 protein levels of the study participants according to immune status can be seen in Table 2. As shown in the table, there were significantly lower serum vitamin D receptor levels and significantly higher HMGB1 protein serum levels in the HIV group with severe immune deficiency (SID-HIV) than the HIV group with mild immune deficiency (MID-HIV) and the healthy control (HC) group.

**Table 2:** Difference of serum VDR and HMGB1 protein levels in the study groups.

Parameters	Group	n	Mean	Standard Deviation	Min	Max	*p*-value
VDR levels (ng/ml)	HC	13	50.05	3.64	44.78	55.57	
	MID-HIV	13	33.72	1.69	31.02	36.23	<0.0001
	SID-HIV	13	25.89	3.95	19.11	30.27	
	Total	39	36.55	9.28	31.63	40.69	
HMGB1 protein levels (pg/ml)	HC	13	680,82	365.51	79.09	1186.32	
	MID-HIV	13	1553.55	231.08	1243.83	1948.43	<0.0001
	SID-HIV	13	3119.81	292.38	2653.03	3573.31	
	Total	39	1784.72	296.32	1325.31	2236,02	

HC: Healthy Control, MID-HIV: Mild Immunodeficiency-HIV patients, SID-HIV: Severe Immunodeficiency-HIV patients, HIV: Human immunodeficiency Virus, BMI: Body Mass Index.

**Fig. 1:**
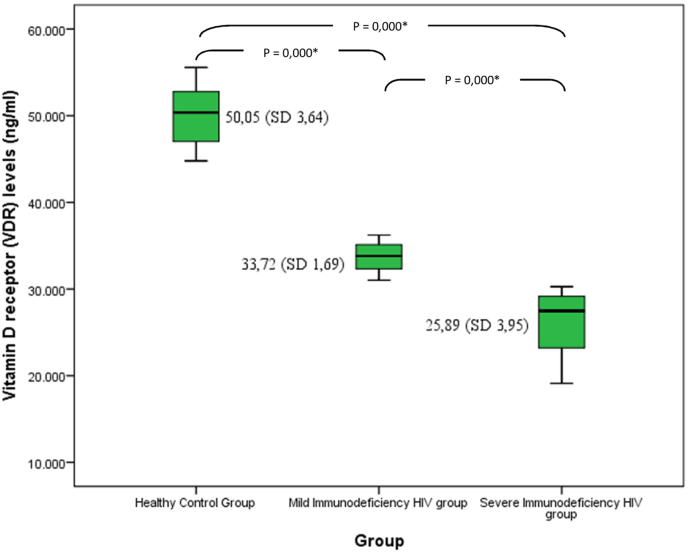
**Mean comparison of vitamin D receptor/VDR levels** in the SID HIV group, MID HIV group and HC group. The box plots show significantly lower level of VDR in the SID group. Non parametric test with Kruskal Wallis was used to compare VDR level (*p* = 0,000, *p* < 0,05), followed by post hoc Mann-Whitney to compare VDR levels between pairs of groups tested (*p* = 0,000 for each groups compared*). HC: Healthy Control, MID: Mild Immunodeficiency, SID: Severe Immunodeficiency, HIV: Human immunodeficiency Virus.

Serum vitamin D receptor level was lower in the SID HIV group than the other 2 groups. As seen in Fig. 1, there was a significant difference (*p* < 0.05) between serum vitamin D receptor levels (25(OH)D) in the SID HIV group (25.89 ± 3.95 ng/ml) than in MID-HIV group (33.72 ± 1.69 ng/ml) and healthy control group (50.05 ± 3.64) ng/ml).

**Fig. 2:**
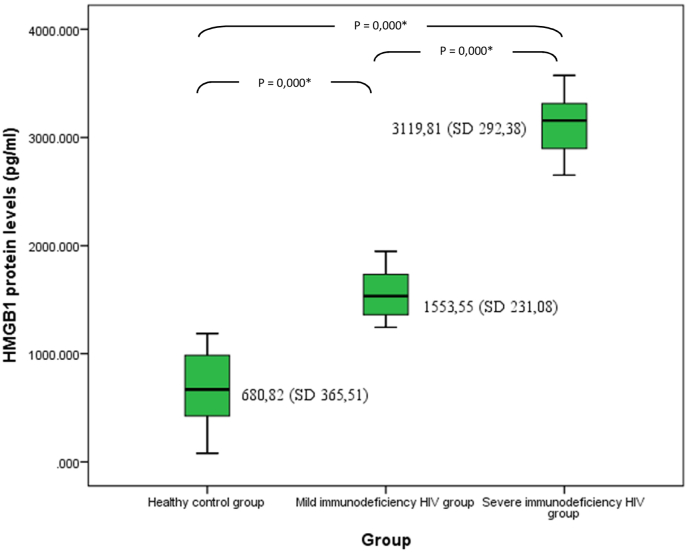
**Mean comparison of High Mobility Group Box 1 (HMGB1) levels** in the SID HIV group, MID HIV group and HC group. The box plots show significantly higher level of VDR in the SID group. Non parametric test with Kruskal Wallis was used to compare VDR level (*p* = 0,000, *p* < 0,05), followed by post hoc Mann-Whitney to compare VDR levels between pairs of groups tested (*p* = 0,000 for each groups compared*). HC: Healthy Control, MID: Mild Immunodeficiency, SID: Severe Immunodeficiency, HIV: Human immunodeficiency Virus.

There was a significant difference (*p* < 0.05) between levels of HMGB1 protein in SID-HIV group (3119.81 ± SD 292.38 pg/ml) compared to those in MID-HIV (1553.55 ± SD 231.08 pg/ml) and healthy control (680.82 ± 365.51 pg/ml). These results indicated an increase in the levels of HMGB1 protein in the SID HIV group compared to the other 2 groups (Fig. 2).

As shown in Table 3, there was a strong negative correlation between vitamin D receptor serum levels and HMGB1 protein serum levels in HIV-infected patients with a correlation coefficient r = −0,932. Meanwhile, the results also showed a strong positive correlation between serum vitamin D receptor levels with immune status with a correlation coefficient r = 0,686 (Table 4).

**Table 3:** Correlation between serum VDR and HMGB1 protein levels in HIV-infected patients.

Variable correlation	HIV infection	
	r*	*p*-value**
Serum VDR and HMGB1 protein levels	−0,932	*p* < 0,01

*r = coefficient correlation. **Pearson correlation test.

**Table 4:** Correlation between serum VDR and HMGB1 protein levels and immune status of HIV-infected patients.

Correlation between variables in HIV-infected patients		
	r*	*p-*value**
1. Seum VDR levels and immune status	0,686	*p* < 0,01
2. Serum HMGB1 protein levels and immune status	−0,816	*p* > 0,05

*r = coefficient contingency. **Chi Square test.

Comorbidities correlation with VDR and HMGB1 were observed and showed at Table 5. There were moderate correlation between anemia with VDR and HMGB1 (r = 0,518, r = −0,518; *p* < 0,001).

**Table 5:** Comorbidities correlation with Vitamin D Receptor and HMGB1 level.

Variable correlation	VDR	HMGB1	*p*-value
	r	r	
1. Hypertension	−0,300	0,300	0,064
2. Diabetes Mellitus	−0,058	0,043	0,727
3. Hepatitis B	−0,202	0,202	0,218
4. Renal insufficiency	−0,259	0,259	0,111
5. Cardiovascular Disease	−0,300	0,300	0,064
6. Anemia	−**0,518**	**0,518**	0,001*

r = coefficient correlation, *p-*value using Spearman test, *p-value < 0,01.

## 4 Discussion

The prevalence of hypovitaminosis D varies from 42 to 95% in different HIV study groups [13]. This study found lower vitamin D receptors in the severe immune deficiency group. We observed mean serum vitamin D receptor levels significantly lower in the SID-HIV group than those in the MID-HIV group and healthy control. This result in accordance with a cohort prospective study of 204 advanced stage HIV-infected women in the US during 24-month observation. The study showed low vitamin D level was associated with decreased CD4 recovery after ARV therapy in HIV patients with severe immune deficiency. Impaired naive CD4 cell production was associated with low vitamin D levels during immune reconstitution [14].

The active form of vitamin D has a significant immunomodulatory effect as an important determinant of CD4 effector T cell differentiation. The biological effect of 1,25(OH)_2_D is mediated by VDR and related to VDR protein expression level. It is known that 1,25(OH)_2_D induces intracellular redistribution of VDR significantly and stabilized VDR levels by protecting against proteasomal degradation. Proteasomal inhibition causes up-regulation of VDR protein and increased activation of genes induced by 1,25(OH)_2_D [15].

There was a significantly strong correlation between serum vitamin D receptor levels and immune status in this study. Vitamin D deficiency contributes to the pathogenesis of HIV infection by disrupting innate and adaptive immune responses. Vitamin D and VDR axis have a strong immunomodulatory effect and antimicrobial effect in HIV infection [7]. Low levels of vitamin D trigger inflammation and the immune system's activation, which will increase the risk of comorbidity and mortality in the HIV-infected population [16]. Havers et al. stated that low 25(OH)D levels were independently associated with higher clinical progression [7]. The important role of vitamin D in host resistance to infection is supported by findings such as 1) immune cells can produce CYP27B1 and convert 25 (OH)D to 1α-25 (OH)_2_D; 2) Most cells of the immune system express VDR; 3) Production of 1α-25 (OH)_2_D from the immune system induces the production of anti-bacterial (cathelicidin, beta-defensin); and 4) Impaired vitamin D status contributes to the burden of infectious diseases worldwide [17,18]. It is known that vitamin D triggers autophagy in macrophages, which significantly inhibits HIV-1 replication but depends on the dose used [19].

We found that the mean of HMGB1 protein levels was higher in the SID-HIV group than the MID-HIV group and healthy controls. There was a robust negative correlation between serum HMGB1 protein levels with immune status (r = −0.816), but it was not significant (*p* > 0,05). Increased HMGB1 was associated with a higher rate of inflammatory conditions in HIV patients and was further associated with immune deficiency conditions. This result in accordance with the retrospective study of 42 HIV-1 infected patients showing increased HMGB1 levels and decreased after effective ARV therapy [20]. The higher levels of HMGB1 protein in people with SID-HIV were associated with HIV infection that attacks primarily the immune system and characterized by the failure of immune cells due to chronic immune activation. The progression of HIV infection is characterized by an increase in enterocyte damage, microbial translocation, and chronic immune activation [21]. In the process of viral infection, HMGB1 is actively or passively released by the infected cells and initiates an inflammatory response driven by the infected cells and the adjacent innate immune cells [22].

HMGB1 is a protein that mediates HIV-1 replication in vitro. It is known that bacterial products and HMGB1 will form an active complex that produces pro-inflammatory conditions, directly promotes viral replication in infected cells, and contributes to the immune system's activation [9,23]. HMGB1 is released by extracellular damage or necrosis, which can act as a potent pro-inflammatory marker by stimulating cytokine expression in monocytes and endothelium [24,25]. HMGB1 can be a natural link between microbial and hyperinflammatory products [8].

There was a significantly strong negative correlation between serum vitamin D receptor levels and HMGB1 protein levels (r = −0.932). This result was consistent with an animal study that found LPS-induced HMGB1 secretion was hindered by 1,25(OH)_2_D via inhibition of HMGB1 translocation from the nucleus to the cytoplasm in macrophages. It is known that 1,25(OH)_2_D3 can induce oxygenase 1, which plays an important role in inhibiting the translocation of HMGB1 in the nucleus and its secretions [10]. A different result from other animal study stated that HMGB1 level did not differ based on vitamin D status and cell type; also, vitamin D has no direct effect on HMGB1 [24].

In this study, low levels of vitamin D receptor in SID-HIV patients could occur through a chronic inflammatory process associated with severe immunodeficiency. Inflammatory causes renal alpha-hydroxylase activity disruption and decreases the production of 1.25(OH)D by inhibiting the conversion of 25(OH)D to 1,25(OH)_2_D, which is stimulated by the PTH hormone [26]. Decreased 1,25(OH)_2_D could decrease induction of vitamin D receptor, resulting in low levels of VDR. Interestingly, the aggravation of the inflammatory factor as a cause can be seen from the increase in HMGB1 levels, which were higher in the SID-HIV group compared with the MID-HIV group and healthy controls. Efavirenz (NNRTI) that is known to influence catabolism of 25(OH)D was used in all this study groups (MID-HIV and SID-HIV) but lower levels of VDR were found in the group with SID-HIV. We found that chronic inflammatory factors and decreased immunity were the main causes of decreased serum VDR which were confirmed by high levels of HMGB1 protein and low levels of CD4.

We observed comorbidities in this study such as hypertension, diabetes mellitus, hepatitis B, mild renal infections, cardiovascular disease, and anemia. We did not find a significant correlation between several comorbidities with VDR and HMGB1 levels from correlation analysis, except for anemia.

The prevalence of anemia in this study was 15.3% in HIV-infected subjects, all of whom were in the SID HIV group. This study found a significant moderate correlation between anemia with VDR and HMGB1 in HIV infection. A study has previously reported the association between Vitamin D and anemia in various healthy and disease populations. The mechanism underlying this association involves reducing proinflammatory cytokines by vitamin D and the direct suppression of hepcidin mRNA transcription [27]. However, there was no previous study that reported the association between anemia with VDR and HMGB1 in HIV infection.

Despite the small sample size and cross-sectional design, this is the first study that found a strong correlation between VDR and HMGB1 serum levels in HIV-infected patients. This study also differentiates the sample into three groups based on immune status. Further study with a large sample size RCT with vitamin D intervention is needed to establish the correlation between VDR and HMGB1 in HIV-infected patients.

## 5 Conclusion

We found significantly lower vitamin D receptor levels and a higher level of HMGB1 serum in SID-HIV patients, suggesting chronic inflammatory factors and decreased immunity as the causes. A strong and significant negative correlation between vitamin D receptor and HMGB1 levels and immune status of HIV patients supports the important role of the vitamin D receptor in immune status and severity of the inflammatory process in HIV-infected patients. A further prospective study is needed to confirm the association. Examination of serum vitamin D or VDR and supplementation of vitamin D in HIV patients when insufficiency or deficiency occurred are warranted.

## Ethical approval

All procedure for human experiment has been approved by 10.13039/501100010740Ethics Commission Faculty of Medicine, Hasanuddin University Number: 1209/UN4.6.4.5.31/PP36/2019.

## Author contribution

ISW, MH, RHM and AB wrote the manuscript and participated in the study design. ISW, MH, RHM, AB, M, MNM, ID drafted and revised the manuscript. ISW, MH, RHM and AB performed treatment. ISW, MH, RHM and AB performed the statistical analyses and revised the manuscript. All authors read and approved the final manuscript.

## Consent

The research was conducted ethically in accordance with the World Medical Association Declaration of Helsinki. The patients have given their written informed consent on admission to use their prospective database and files for research work.

## Registration of research studies

1. Name of the registry: research registry.

2. Unique Identifying number or registration ID: researchregistry6352.

3. Hyperlink to your specific registration (must be publicly accessible and will be checked): https://www.researchregistry.com/browse-the-registry#home/?view_2_search=6352&view_2_page=1.

## Guarantor

Indah Sapta Wardani.

## Provenance and peer review

Not commissioned, externally reviewed.

## Funding

No funding or sponsorship.

## Declaration of competing interest

The authors declare that they have no conflict of interests.

## References

[1] UNAIDS (2020). Global AIDS Update 2020.

[2] Kementrian Kesehatan Republik Indonesia (2016). Direktorat Jendral Pencegahan Dan Pengendalian Penyakit. "Laporan Situasi Perkembangan HIV & AIDS di Indonesia Januari - Maret 2016," Kementrian Kesehatan Republik Indonesia.

[3] Pusat Data dan Informasi Kemenkes (2014). Situasi dan analisis HIV AIDS. http://www.depkes.go.id/resources/download/pusdatin/infodatin/Infodatin%20AIDS.pdf.

[4] Alvarez N., Aguilar-Jimenez W., Rugeles M.T. (2019 Sep 25). The potential protective role of vitamin D supplementation on HIV-1 infection. Front. Immunol..

[5] Jiménez-Sousa M.Á., Martínez I., Medrano L.M., Fernández-Rodríguez A., Resino S. (2018). Vitamin D in human immunodeficiency virus infection: influence on immunity and disease. Front. Immunol..

[6] Lang P.O., Aspinall R. (2017 May). Vitamin D status and the host resistance to infections: what it is currently (not) understood. ClinTher.

[7] Havers F., Smeaton L., Gupte N., Detrick B., Bollinger R.C., Hakim J., Kumarasamy N., Andrade A., Christian P., Lama J.R., Campbell T.B., Gupta A. (2014 Jul 15). ACTG PEARLS; NWCS 319 Study Teams. 25-Hydroxyvitamin D insufficiencyanddeficiencyisassociatedwith HIV diseaseprogressionandvirologicalfailurepost-antiretroviraltherapyinitiation in diversemultinationalsettings. J. Infect. Dis..

[8] Troseid M., Sonnerborg A., Nowak P. (2011). High mobility group box protein-1 in HIV-1 infection: connecting microbial translocation, cell death and immune activation. Curr. HIV Res..

[9] Nowak P., Abdurahman S., Lindkvist A., Troseid M., Sonnerborg A. (2012). Impact of HMGB1/TLR ligand complexes on HIV-1 Replication: possible role for flagellin during HIV-1 infection. International Journal of Microbiology.

[10] Barqasho B dkk (2010). Implications of the release of high-mobility group box 1 protein from dying cells during human immunodeficiency virus type 1 infection in vitro. J. Virol..

[11] Rao Z., Zhang N., Xu N., Pan Y., Xiao M., Wu J., Zhou H., Yang S., Chen Y. (2017 Oct 16). 1,25-Dihydroxyvitamin D inhibits LPS-induced high-mobility group box 1 (HMGB1) secretion *via* targeting the NF-E2-Related factor 2-hemeoxygenase-1-HMGB1 pathway in macrophages. Front. Immunol..

[12] Agha R., Abdall-Razak A., Crossley E., Dowlut N., Iosifidis C., Mathew G. (Dec. 2019). STROCSS 2019 Guideline: strengthening the reporting of cohort studies in surgery. Int. J. Surg..

[13] Viard J.-C., Souberbielle O., Kirk (2011). Vitamin D and clinical disease progression in HIV infection: results from the EuroSIDA Study. AIDS.

[14] Aziz M., Livak B., Burke-Miller J. (2013). Vitamin D insufficiency may impair CD4 recovery among Women's Interagency HIV Study participants with advanced disease on HAART. AIDS.

[15] Kongsbak M., von Essen M.R., Boding L. (2014). Vitamin D up-regulates the vitamin D receptor by protecting it from proteasomal degradation in human CD4+ T cells. PloS One.

[16] Watkins R.R., Lemonovich T.L., Salata R.A. (2015). An update on the association of vitamin D deficiency with common infectious diseases. Can. J. Physiol. Pharmacol..

[17] Ross A.C., Judd S., Kumari M., Hileman C., Storer N., Labbato D., etal (2011). Vitamin D islinkedtocarotid intima-media thicknessandimmunereconstitution in HIV-positiveindividuals. Antivir. Ther..

[18] Abhimanyu, Coussens A.K. (2017). The roleof UV radiationand vitamin D in theseasonalityandoutcomesofinfectiousdisease. Photochem. Photobiol. Sci..

[19] Huang S.J., Wang X.H., Liu Z.D. (2016). etal. Vitamin D deficiencyandtheriskoftuberculosis: a meta-analysis. Drug Des DevelTher.

[20] Trøseid M., Nowak P., Nystrom J., Lindkvist A., Abdurahman S., Sonnerborg A. (2010). Elevated plasma levels of lipopolysaccharide and high mobility group box-1 protein areassociated with high viral load in HIV-1 infection: reduction by 2-year antiretroviral therapy. AIDS.

[21] Nowak P., Barqasho B., Sonnerborg A. (2007). Elevated plasma levels of high mobility group box protein 1 in patients with HIV-1 infection. AIDS.

[22] Trøseid M., Lind A., Nowak P. (2013 Jun). Circulating levels of HMGB1 are correlated strongly with MD2 in HIV-infection: possible implication for TLR4-signalling and chronic immune activation. Innate Immun..

[23] Fiuza C., Bustin M., Talwar S. (2003). Inflammation-promoting activity of HMGB1 on human microvascular endothelial cells. Blood.

[24] Nguyen A.H., Lim V.M., Fleegel J.P., Hunter W.J., Agrawal D.K. (2016). Cutaneous expression of TREM, vitamin D receptor and HMGB1 in vitamin D deficiency. Int. J. Clin. Exp. Pathol..

[25] Zhang H., Yang N., Wang T., Dai B., Shang Y. (2018). Vitamin D reduces inflammatory response in asthmatic mice through HMGB1/TLR4/NF-κB signaling pathway. Mol. Med. Rep..

[26] Lake J.E., Adams J.S. (2011). Vitamin D in HIV-Infected patients. Curr. HIV AIDS Rep..

[27] Smith E.M., Tangpricha V. (2015). Vitamin D and anemia: insights into an emerging association. Curr. Opin. Endocrinol. Diabetes Obes..

